# Poly(*l*-Lactic Acid)/Pine Wood Bio-Based Composites

**DOI:** 10.3390/ma13173776

**Published:** 2020-08-26

**Authors:** Monika Dobrzyńska-Mizera, Monika Knitter, Aneta Woźniak-Braszak, Mikołaj Baranowski, Tomasz Sterzyński, Maria Laura Di Lorenzo

**Affiliations:** 1 Institute of Materials Technology, Polymer Division, Poznan University of Technology, 61-138 Poznan, Poland; monika.knitter@put.poznan.pl (M.K.); tomasz.sterzynski@put.poznan.pl (T.S.); 2 Department of High Pressure Physics, Faculty of Physics, Adam Mickiewicz University, 61-614 Poznan, Poland; abraszak@amu.edu.pl (A.W.-B.); mikbar@amu.edu.pl (M.B.); 3 National Research Council (CNR), Institute of Polymers, Composites and Biomaterials (IPCB), c/o Comprensorio Olivetti, via Campi Flegrei, 34, 80078 Pozzuoli, NA, Italy; marialaura.dilorenzo@ipcb.cnr.it

**Keywords:** poly(*L*-lactic acid), pine wood, compatibilization, γ-aminopropyltriethoxysilane, bio-based composite

## Abstract

Bio-based composites made of poly(*l*-lactic acid) (PLLA) and pine wood were prepared by melt extrusion. The composites were compatibilized by impregnation of wood with γ-aminopropyltriethoxysilane (APE). Comparison with non-compatibilized formulation revealed that APE is an efficient compatibilizer for PLLA/wood composites. Pine wood particles dispersed within PLLA act as nucleating agents able to start the growth of PLLA crystals, resulting in a faster crystallization rate and increased crystal fraction. Moreover, the composites have a slightly lower thermal stability compared to PLLA, proportional to filler content, due to the lower thermal stability of wood. Molecular dynamics was investigated using the solid-state ^1^H NMR technique, which revealed restrictions in the mobility of polymer chains upon the addition of wood, as well as enhanced interfacial adhesion between the filler and matrix in the composites compatibilized with APE. The enhanced interfacial adhesion in silane-treated composites was also proved by scanning electron microscopy and resulted in slightly improved deformability and impact resistance of the composites.

## 1. Introduction

Application of natural fiber reinforced composites increases in all areas of production, especially the building and automotive industry. They are usually based on commodity polymers, like polypropylene or polyvinyl chloride. However, with an increasing environmental awareness there is a need to seek and replace traditional composites, which are difficult to recycle, with bio-based polymers and their composites, which can be easily disposed at their end of lifetime [1].

Several bio-based and/or biodegradable polymers have been developed commercially, such as polyhydroxyalcanoates, poly(*l*-lactic acid) (PLLA), polycaprolactone and polybutylene succinate and their derivates [2]. Among those listed, PLLA gained the greatest attention due to its numerous advantages such as being biodegradable and compostable, having good stiffness and strength and being able to be produced on a large scale by microbial fermentation of agricultural byproducts [1,3,4]. Besides its advantages, PLLA has also some drawbacks, mainly brittleness and slow crystallization kinetics that should be overcome to further develop its application possibilities [5,6,7]. Several attempts have been made to outbalance these disadvantages, which mainly include the modification of PLLA formulations to develop material with improved plasticization [6,8], impact resistance [1,9], or crystallization rate [10], often achieved by introduction of particles and fiber-like fillers [11,12,13].

The incorporation of natural fibers into PLLA results in price reduction, as natural fibers are typically industrial biowaste. Previous literature studies indicate that the addition of natural fibers to PLLA leads to compostable materials with increased stiffness and even an accelerated biodegradation process due to faster hydrolysis and oxidation of the polymer matrix and filler in specific environment [14,15]. During the last two decades, researchers extensively studied PLLA-based composites attained with a vast variety of natural fibers, such as: wood [16], cotton [17], nut shells [15], bamboo [18], cereal straw [19], jute [20], sisal [21], and abaca [22]. Recently, attention has been focused on PLLA/wood composites, due to their promising properties and potential applications [19]. The type of wood, namely its specific chemical composition, may affect the possible interaction with the matrix [23]. Pine wood, which is the filler selected for our study, is mainly composed of cellulose (40–50 wt %), lignin (25–30 wt %), hemicellulose (20–25 wt %), and small amounts of fatty acids and resins (up to 10 wt %) [24]. The building blocks of pine wood are microfibrils made of highly crystalline cellulose acting as the frame, surrounded by semicrystalline hemicellulose as the matrix and covered by amorphous lignin. These microfibrils have a diameter of 10–20 nm and make up the cell walls of the tree [24].

Previous literature studies indicate the need to improve interfacial adhesion between the wood filler and PLLA. Wood particles have often a large size, of the order of hundreds of microns, which easily debond from the polymeric matrix under external load [1,25,26]. Debonding causes formation of voids and in turn premature failure of the material, as it was demonstrated for instance in the deformation process of polypropylene/wood composites not containing any coupling agent [25,26]. A number of coupling agents were tested to improve interfacial adhesion between PLLA and wood, with only limited results in terms of improved compatibility, as for *N*,*N*-(1,3-phenylene dimaleimide) and 1,1-(methylenedi-4,1-phenylene)bismaleimide [26], phenolic resin [14], or bioadimide [11].

Silane coupling agents were also tested, with efficiency found to be affected by the specific functional groups. γ-methacryloxypropyltrimethoxysilane was used as a compatibilizer in PLLA/pine wood flour composites, with poor results in terms of even increased brittleness compared to PLLA [27,28]. An improvement of the thermal and mechanical properties of PLLA reinforced with Stika fibers treated with vinyltrimethoxysilane was reported [29]. A direct comparison of the efficiency of silane functional groups was conducted for unsaturated polyester and epoxy resin matrices filled with silane-treated cellulose fibers, using γ-aminopropyltriethoxysilane (APE), γ-methacrylopropyltrimethoxysilane, hexadecytrimethoxysilane, and γ-mercaptopropyltrimethoxysilane as coupling agents [30]. An increase of the modulus and tensile strength in the resulted composites was observed, with the strongest reinforcing effect found for APE-treated fibers, and rationalized with higher reactivity of the (–NH_2_) functions [27,28]. APE was also found to be an efficient coating agent in PLLA/cellulose fibers and nanofibers composites, by promoting dispersion of cellulose nanofibers [31], with improved adhesion between the phases and increased storage modulus in PLLA/cellulose fibers composites [28]. APE could also promote compatibilization between PLLA and lignin, the other major constituent of pine wood, when used as coating for lignin [32,33]. These literature results suggest high potential for APE to act as an efficient coupling agent also for PLLA composites containing pine wood, where, as mentioned above, cellulose and lignin are the major constituents. To our knowledge, to date APE has not been tested yet as a compatibilizer for PLLA/pine wood composites.

Numerous studies have been conducted on PLLA/wood blends in order to investigate their molecular structure, crystallization kinetics, thermal, and mechanical properties [16,17,18,19,20,21,22,34]. Researchers also focused on testing different PLLA formulations using solid-state NMR techniques, mainly high-resolution ^13^C and ^1^H NMR spectra and carbon spin-lattice relaxation times *T_1_* (^13^C) [35,36,37,38,39,40,41,42,43,44,45,46]. However, according to the best of our knowledge, very limited research describes molecular dynamics of PLLA-based modifications using the temperature studies of spin-lattice relaxation times (*T_1_*) [47]. This type of the solid-state NMR technique describes molecular dynamics of local and segmental motions of side groups and polymer chain segments and allows for determination of diffusion coefficients, which provide information on the size of domains in heterogeneous systems [48]. The results presented herein provide important insights into relaxation processes of entire PLLA chains. Motions of polymer chain segments and side groups may be significantly influenced by the presence of a filler and/or compatibilizer, as they may interact via functional groups of PLLA, such as carbonyl (–CO), methine (–CH) and methyl (–CH_3_), with numerous side groups of lignin and cellulose, i.e., methoxyl groups (–OCH_3_), phenolic and aliphatic hydroxyl groups (–OH), and amino functionals (–NH_2_) of the APE binder [28,32,33,49].

The aim of this work is to study the influence of pine wood surface-modified with APE, on structure and mechanical properties of PLLA/wood composites, with the final objective of producing completely biodegradable and bio-based wood-polymer composites.

## 2. Experimental

### 2.1. Materials

A commercial poly(lactic acid) 3100HP, abbreviated PLLA, with MFR 24 g/10 min (210 °C, 2.16 kg) produced by Nature Works (Blair, NE, USA) was used. Pine wood particles were kindly obtained from Wood Technology Institute in Poznan, Poland. The as-received particles were initially selected using a Fritsch Analysette 3 vibratory sieve shaker to have a uniform diameter distribution, then their size was measured with an Opta-Tech optical microscope (Warsaw, Poland), model MB200 combined with an Opta-Tech MI6 camera (Warsaw, Poland) and a Opta-Tech Capture 2.0 software. The used particles have a length (*L*) of 1.25 ± 0.63 mm and diameter (*D*) of 0.47 ± 0.15 mm, and an average aspect ratio (*L/D*) of 2.6. Composition of pine wood particles was determined by thermogravimetry, using the procedure detailed in Ref. [50,51,52,53]: the pine wood particles consisted of 62 wt % of hemicellulose and cellulose, 34 wt % of lignin and the remaining part (4 wt %) were fatty acids and resins. The chemical coupling agent was 3-aminopropylthrietoxysilane (APE) produced by UniSil (Poland), which aimed to promote adhesion between the polymer and filler.

### 2.2. Wood Impregnation with Compatibilizer

APE was dissolved in ethanol (3% v/v), then the proper amount of wood to attain impregnation with 3 wt % of APE was added to the system. The filler was transferred slowly into the APE solution and stirred for 1 h at 2000 rpm. The whole impregnation procedure was conducted at room temperature. The wood filler was then filtered and dried under vacuum at 80 °C for 24 h.

### 2.3. Composites Preparation

The composites were prepared following a four-step procedure. At first, the wood filler was modified by impregnation with APE, then the solid-state mixed with PLLA pellets, then melt mixed with the polymer matrix and finally shaped to obtain plates by compression molding. All materials were dried prior to processing at 80 °C for 3 h. Poly(lactide acid) pellets were mixed with the pine wood filler in a rotary mixer Retsch GM 200 (Haan, Germany) for 3 min at a rotation speed of 2000 rpm. Homogenization of the premixed materials with different wood contents (0–30 wt %) and compatibilizer concentration of either 0 or 3 wt % to the wood content was ensured by molten state extrusion with a Zamak corotating twin screw extruder operated at 180 °C and 100 rpm. The extruded rod was cooled in air and pelletized. PLLA/wood and PLLA/wood/APE composites at various compositions were prepared, as summarized in Table 1.

The composites were compression-molded with a Remi-Plast Laboratory Forming Press PLLHS-7 (Czerwonak, Poland) at a temperature of 185 °C for 3 min, without any pressure applied, to allow complete melting. After this period, a load of about 175 bar was applied for 3 min, and then the samples with a thickness of 1 mm were cooled in air to room temperature.

### 2.4. Methodology

#### 2.4.1. Differential Scanning Calorimetry (DSC)

The thermal properties of PLLA and PLLA composites were investigated with a Netzsch DSC 204 F1 Phoenix^®^ apparatus (Selb, Germany), using aluminum crucibles and samples of approximately 3 mg, under nitrogen flow. The instrument was calibrated in terms of temperature by analysis of high purity standards including indium, tin, bismuth, zinc, and aluminum, while the energy calibration was performed by analysis of the melting enthalpy of indium. All the samples were heated to 200 °C and held in a molten state for 5 min, followed by cooling to 20 °C. Heating rates were 10 K·min^−1^, whereas a lower cooling rate of 5 K·min^−1^ was preferred, due to the low crystallization kinetics of PLLA [4]. The first run of DSC curves, revealing the thermal history of the materials, was evaluated to gain broad information on the PLLA matrix modification. The glass transition temperature (*T*_g_), melting temperature (*T*_m_), cold crystallization temperature (*T_cc_*), crystallization temperature (*T*_c_), and heat of melting (Δ*H*_m_) were determined for all the samples.

#### 2.4.2. Wide-Angle X-ray Diffraction (WAXD)

Wide-angle X-ray diffraction (WAXD) measurements were performed on a VEB TuR, model TUR–M62 diffractometer with CuK_α_ radiation (Dresden, Germany). The scanned 2θ range was from 1 to 30° with a scanning rate of 0.01° and time per step of 5 s. The samples used for the WAXD analysis were in a form of compression molded sheets of 1.0 mm thickness. Characteristic peaks were assigned according to the literature [3]. The crystallinity degree (*Χ_c_*) of PLLA formulations was evaluated as the ratio between the diffraction due to the crystalline phase (*A_c_*) divided by the total diffraction intensity (*A_c_ + A_a_*) [54,55]:

(1) $${Χ}_{c}=\frac{A_{c}}{A_{c}+A_{a}}$$

where: *A_c_* is the crystalline phase and *A_a_* is the amorphous phase.

#### 2.4.3. Solid-State ^1^H NMR

Solid-state ^1^H NMR measurements of the spin-lattice relaxation times *T_1_* in the laboratory frame were performed on a pulse spectrometer (Poznan, Poland) operated at 30.2 MHz [47,56]. The samples of PLLA with various amounts of wood were sealed in the glass tubes after being degassed to avoid humidity effects and to remove paramagnetic oxygen. The spin-lattice relaxation times *T_1_* were measured with the conventional saturation recovery sequence ended with a solid echo within the temperature range from −150 to 125 °C (123–393 K).

The spin-relaxation time (T_1_) was estimated from fitting the biexponential equation [57]:

(2) $$\frac{M_{0}-M_{z}\left(t\right)}{M_{0}}=A1exp\left(-\frac{t}{T_{1L}}\right)+A2exp\left(-\frac{t}{T_{1S}}\right)$$

where: *M_o_* is the equilibrium magnetization, *T_1L_* is the long and *T_1S_* is short relaxation time, and *A1* and *A2* are magnetization fractions of long and short component, respectively.

In case of the PLLA/30 W sample, the recovery of the magnetization was three-exponential resulting in three magnetization fractions that needed to be considered.

The activation parameters describing molecular dynamics of PLLA formulations were determined by analyzing the temperature dependence of spin-relaxation times *T_1_* basing on the dipole–dipole Bloembergen–Purcell–Pound (BPP) theory [58]. It was assumed that below the glass transition temperature, *T_1_* values were caused by dipolar interactions modulated by local motions of both molecular groups and chain segments. As these contributions are additive, *T_1_* was determined from the following Equation (3) [58,59,60,61]:

(3) $$\frac{1}{T_{1}}=\frac{2}{3}\gamma^{2}\sum_{k}\Delta M_{2k}\left[J_{k}\left(\omega \right)+4J_{k}\left(2\omega \right)\right]$$

where: γ is a gyromagnetic ratio of protons, ΔM_2k_ is a reduction of the second moment in ^1^H NMR spectra induced by motions in the studied system, and J_k_(*ω*) denotes the spectral density function for the angular frequency *ω*.

Due to a very broad and asymmetrical in shape minima of relaxation times *T_1_* in polymeric systems, a distribution of correlation times was needed to describe the relaxation processes. Spectral density function, given by Davidson and Cole, was used to determine J(*ω*) according to formula (4) in Equation (3) [62]

(4) $$J\left(\omega ,\tau_{c},\beta \right)=\frac{2}{\omega }\left[\frac{sin\left(\beta arctan\left(\omega \tau_{c}\right)\right)}{{\left(1+\omega^{2}\tau_{c}^{2}\right)}^{\frac{\beta }{2}}}\right],$$

where: *τ_c_* is the upper cut-off correlation time and *β* is the distribution width of correlation times. Assuming that motions are thermally activated, the temperature dependence of the correlation time *τ_c_* can be expressed by the Arrhenius formula (5):

(5) $$\tau_{c}=\tau_{0}exp\left(\frac{E_{a}}{RT}\right),$$

where: *τ_0_* is the pre-exponential factor, *E_a_* is the activation energy of molecular motion, and *R* is the universal gas constant.

#### 2.4.4. Thermogravimetry (TGA)

Thermostability was determined by thermogravimetric analyses (TGA) with temperatures set between 30 and 600 °C, at a heating rate of 10 °C·min^−1^, under the nitrogen atmosphere using a Netzsch TG 209 F1 apparatus (Selb, Germany) The instrument was temperature-calibrated by analysis of high purity standards including indium, tin, bismuth, zinc, aluminum, and silver, while the balance was calibrated automatically with an inbuilt calibration weight. Approximately 10 mg samples were placed in ceramic pans. The decomposition onset temperature *T_o_* was determined at the point of intersection of tangents to two branches of the thermogravimetric curve [63]. The residual mass (*m_R_*) of each sample was determined at 600 °C. Before the tests, a blank curve was measured and subtracted from the single thermograms, to correct from instrumental drift.

#### 2.4.5. Scanning Electron Microscopy (SEM)

Morphological analysis of fractured PLLA/wood and PLLA/wood/APE composites was performed using a Jeol 7001TTLS scanning electron microscope (SEM; Boston, MA, USA) using an accelerating voltage of 10–20 kV. Before analysis, the samples were sputtered-coated with gold, then mounted on aluminum stubs.

#### 2.4.6. Mechanical Properties

Composite tensile analysis was carried out by single-axis extension (crosshead speed 50 mm min^-1^) using an Instron model 4481 with the self-clamping action grip and 50 kN head capacity (Norwood, MA, USA). Following ISO 527-1 the Young’s modulus and elongation at break were determined [64]. The tensile impact strength of the unnotched samples with 10 mm × 4 mm × 80 mm dimension were determined in accordance with ISO 8256, using hammer type PSD50/15 [65]. Ten specimens were tested in each case in order to ensure measurement reproducibility.

## 3. Results and Discussion

**Figure 1:**
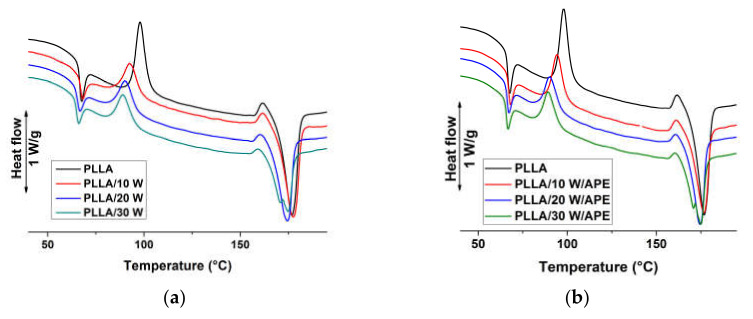
Heat flow rate plots of PLLA and PLLA/wood composites of the indicated compositions upon heating at 10 K/min. (**a**): PLLA/wood composites and (**b**): PLLA/wood/APE composites. Exotherm upward.

**Figure 2:**
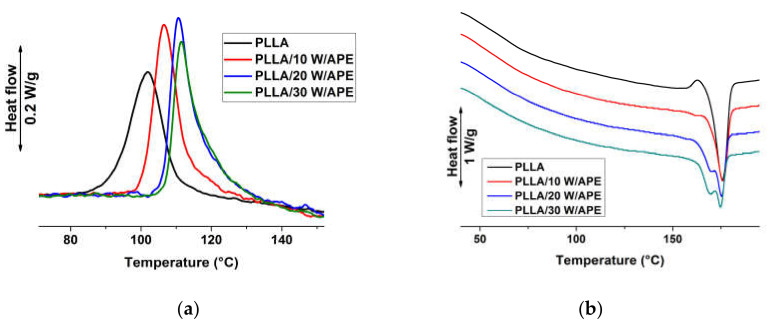
Heat flow rate plots of PLLA and PLLA/wood composites of the indicated compositions upon cooling at 5 K/min (**a**) and upon subsequent heating at 10 K/min (**b**). Exotherm upward.

Heat flow rate plots of PLLA and PLLA/wood compression-molded composites are presented in Figure 1 and Figure 2. Figure 1 illustrates the heat flow rate plots of compression molded sheets of the composites without (left) and with APE compatibilizer (right). The DSC plot of plain PLLA displays a glass transition (*T*_g_) at 67 °C coupled with sizable enthalpy relaxation, followed by a sharp cold crystallization exotherm peaked at 98 °C, and by a multiple thermal event, typical of PLLA, due to transformation from α’-crystal to α-form [2,3,4]. Comparison of the enthalpy associated to cold crystallization, crystal reorganization, and melting indicates that compression-molded PLLA has a low crystal fraction. Addition of wood, both plain and impregnated with APE, results in a somewhat smaller heat capacity step at *T*_g_, whose temperature does not vary with composition, and in a smaller cold crystallization peak. The latter appears anticipated compared to plain PLLA of a few degrees, with the exact temperature being affected by wood content. Additionally, melting behavior varies with polymer composition, as an expected consequence of the varied crystallization temperature. Similar to the DSC trace of plain PLLA, all the analyzed composites display complex melting behavior, caused by metastability of α’-crystals that transform upon heating to α-modification. Comparison of crystallization and melting peak areas indicates a slightly higher crystal fraction in the composites compared to PLLA, whose quantitative analysis is discussed below. No marked variation in thermal properties appears for compatibilized and non-compatibilized compositions, at the parity of wood content.

The DSC plots gained upon cooling of plain PLLA and of PLLA composites containing wood impregnated with APE are illustrated in the left part of Figure 2. For clarity of presentation, only DSC data of compatibilized compositions are shown, being data of non-compatibilized samples very similar, as also seen in Figure 1. In all analyzed compositions PLLA crystallizes upon cooling at 5 Kmin^−1^, with an exotherm that varies with wood content. Higher amounts of wood result in a faster crystallization, with the onset of phase transition shifted to higher temperatures upon cooling, favored by nucleation of PLLA spherulites on wood particles [66,67]. However, the nucleation effect is leveled off at high wood content, as proven by the almost overlapping plots of the composites containing 20 and 30 wt % of compatibilized wood shown in Figure 2. The melting behavior of the samples crystallized during cooling at 5 Kmin^−1^ is presented in the right part of Figure 2. All analyzed compositions display multiple melting, as also seen in Figure 1, with more evident exotherm preceding the final melting seen in plain PLLA, due to lower crystallization temperature and in turn, larger α’-fraction. Main data derived from the DSC analysis of Figure 1 and Figure 2 are summarized in Table 2 together with crystallinity value (*X_c_*) gained from WAXD analysis.

**Figure 3:**
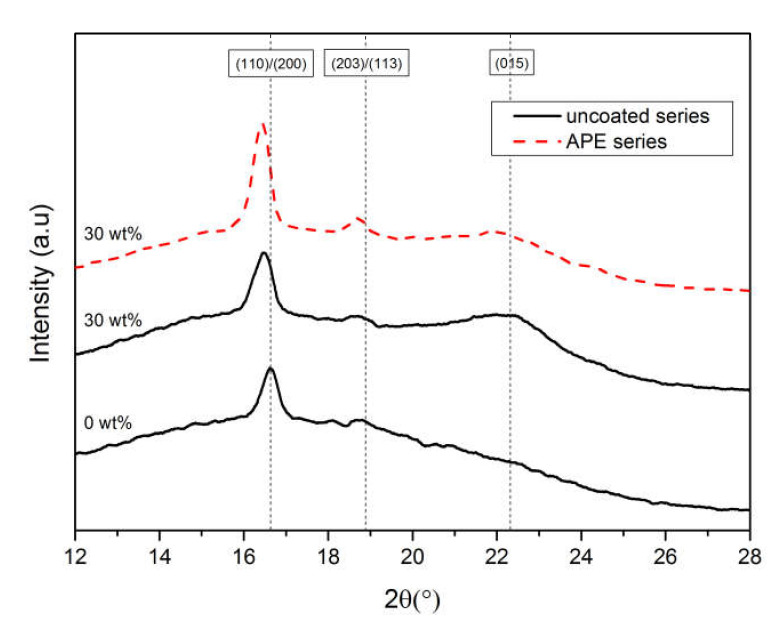
Instrumental-background corrected wide-angle X-ray diffraction (WAXD) patterns of compression molded sheets of PLLA and composites filled with 30 wt %. Symbols: PLLA/wood (▬); PLLA/wood/APE (**---**).

Crystal fraction of plain PLLA and of the composites was quantified by WAXD analysis. Experimental data are exampled in Figure 3 for the plain polymer and the composites containing 30 wt % of filler. The other compositions display WAXD patterns that are similar to those of the composite with lower wood content. The main reflections are indexed in Figure 3. Pure PLLA reveals two strong scattering peaks, at wavelengths of 16.6 and 18.8°, associated with the (110)/(200) and (203) lattice planes respectively, whose position shifts due to a varied ratio between α’- and α-crystals formed during crystallization of compression molded sheets [3,54,68,69]. In the composites, a shift of the above-mentioned lattice planes towards lower 2θ values was noted and assigned to a higher ratio of α’-crystals compared to the plain polymer. Moreover, in case of each wood-reinforced formulation, besides the two main peaks shifted towards lower 2θ values, an additional scattering peak at 22.2°, associated with the (015) lattice plane, can be noticed. This peak, not observed in plain PLLA, indicates the presence of α-modification [54] developed upon cooling of the compression molded composites, together with α’-crystals whose presence is revealed by the position of (110)/(200) reflection. WAXD data indicate that all compression-molded samples start to crystallize at temperatures where α-crystals predominate, with a crystallization tail in the composites that extends to the temperature ranges of α’-crystals growth.

Analysis of WAXD patterns allowed us to calculate the crystal fraction of PLLA and of the composites, using Equation (1), with data shown in Table 2. Crystallinity values determined by WAXD are in good agreement with those determined by the DSC plots of Figure 1, despite a precise calculation by the integration of DSC exothermic and endothermic peaks is complicated by phase transitions of α’- to α-crystals, whose melting and crystallization enthalpy are largely different [70]. Crystallinity degree values were normalized to PLLA content. Pure PLLA reveals *Χ_c_* = 5%. Addition of uncoated wood causes a gradual increase in this value up to 12% for PLLA/30 W, whereas in the case of the compatibilized series this rise is slightly more pronounced resulting in a value of 14% for the highest wood concentration, with the small differences close to experimental uncertainty. The increased crystal fraction in the composites, compared to the plain polymer, confirms nucleation efficiency of the filler, as also seen by the DSC analysis.

**Figure 4:**
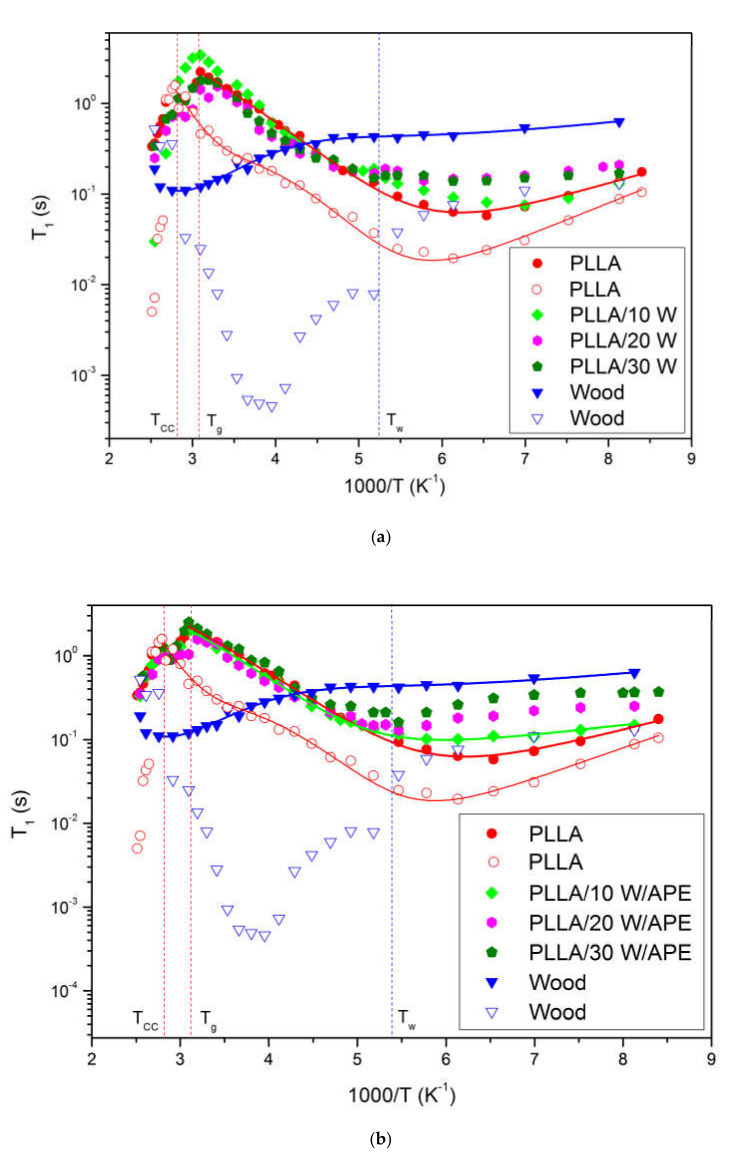
The temperature dependence of spin-relaxation time *T_1_* for (**a**) PLLA/W and (**b**) PLLA/W/APE samples. The solid lines are example fits of Equation (2) with experimental data. Dashed lines emphasize phase transitions occurring in the systems (cold crystallization temperature *T_cc_*, glass temperature *T_g_,* and the phase transition occurring in wood *T_w_*). Long *T_1L_* and short *T_1S_* components of relaxation times are marked with full and empty symbols, respectively.

Solid-state ^1^H NMR spectroscopy was employed to probe the effect of natural fibers addition on the molecular mobility of PLLA chains. The temperature dependence of the spin-lattice relaxation times, *T_1_*, is presented in Figure 4a,b and provides information on variations of the relaxation processes and microstructure of the PLLA composites. As shown by the WAXD analysis, the PLLA consists of a crystalline and amorphous phase, hence it is assumed that the PLLA heterogeneity causes nonexponential magnetization recovery [41,71,72]. When a system consists of small number separated phases with different mobilities and the spin diffusion between their protons in different phases is not effective, multiple exponential spin-lattice relaxation was observed. The ^1^H magnetization recoveries for all the PLLA samples were analyzed as a two-exponents sum, which indicated an occurrence of two phases with different *T_1_*’s. The long *T_1L_* and short *T_1S_* components of relaxation times indicate the existence of different phases with various chain mobilities [40]. Only for the PLLA/30 W sample, the magnetization recovery was found to be three-exponential, which proves the presence of three fractions. In a heterogeneous system, different phases can relax with its own relaxation time *T_1_* [43].

Pure PLLA, as depicted in Figure 4a, revealed biexponential magnetization recovery. The ratio of the magnetization fractions associated with *T_1L_* and *T_1S_* was 7/3 throughout the tested temperature range. These two relaxation times possess similar reciprocal temperature dependence with one broad and asymmetrical minimum in the low temperature range, corresponding to molecular processes occurring in PLLA. It is assumed that the long relaxation time *T_1L_* is associated with the rigid amorphous phase in the sample, while the *T_1S_* fraction is related to the more mobile amorphous phase. According to previous NMR studies [46,61,73,74,75,76] the broad minimum at a low temperature of about −140 °C (133 K) might be associated with hindered rotations of nonequivalent CH_3_ groups. The dielectric spectroscopy analyses indicate that in semicrystalline polymers, among others in PLLA, the secondary relaxation process *β* was observed below *T_g_*, at lower temperatures, ranging between −30 and −150 °C, and represents localized motions of side groups or main polymer chains including thermal motions of carboxyl, hydroxyl or ester functional groups or twisting motions of main polymer chains [77,78,79]. Taking into account all the above, it is assumed that the low temperature minimum of the relaxation times *T_1_*, shown in Figure 4a, may be assigned to local motions of methyl group CH_3_ attached to main PLLA chains. Additionally, below the glass temperature *T_g_* local twisting motions of main polymer chains occurred during their relaxation. In Figure 4a, it is shown that PLLA curves exhibited two discontinuities reflecting two different phase transitions, marked with red dashed lines, which are associated with cold crystallization *T_cc_* and glass transition *T_g_* temperatures, as proved by DSC studies.

The pine wood used as a filler is mainly composed of cellulose, lignin, hemicellulose, and a small amount of fatty acids and resins, as detailed above. The molecular structure of cellulose indicates a presence of a large number of hydroxyl groups that are responsible for the formation of intramolecular and intermolecular hydrogen bonds [73]. The cellulose and its derivatives are built from glucose units linked to polymer chains via oxygen atoms creating a glucosidic linkage. The constitutive unit of the cellulose contains two hydroxyl groups (–OH) and a methylol group (–CH_2_OH) [76]. Dielectric studies [77,78,79] proved that at low temperatures, below the glass transition, cellulose and its derivatives distinguish two relaxation processes, i.e., the *β* relaxation process associated with local motions of cellulose chains via glucosidic bond with the activation energy *E_a_* ranging between 36 and 51 kJ/mol, and *γ* process related to methylol and hydroxyl side-group motions with activation energy *E_a_* of 9.2 kJ/mol. Lignin is amorphous, highly branched phenolic polymer with multiple functional groups, such as: methoxyl, carbonyl, carboxyl, and hydroxyl linked with aromatic or aliphatic moieties, composed of various amounts and proportions, leading to different compositions and structures [80]. The NMR data of neat wood are collected in Figure 4a,b. Two components of relaxation times, *T_1_*_L_ and *T_1S_*, proved a presence of the rigid and mobile phase in the pine wood structure. In case of the long component of relaxation times *T_1L_*, the temperature dependence of spin-lattice relaxation times revealed a broad and very shallow minimum at low temperatures and a significantly deeper one at the high temperatures’ region. Taking into account temperature values at which minima appeared, it may be assumed that the low temperature minimum occurred due to molecular motion of nonequivalent methyl groups CH_3_ and the high temperature minimum was associated with segmental motions of polysaccharide chains [81,82,83,84]. For the short component relaxation times *T_1S_*, in a range from −150 °C (123 K) to a temperature of about −23 °C (250 K), a gradual decrease in this value occurred, however it did not achieve the minimum due to phase transition *T_w_*, as marked with a dashed line in Figure 4a. Then, an increase of the relaxation times was observed. It is worth pointing out that the phase transition *T_w_* occurred at a temperature of −83 °C (190 K). Wood particles used in the study contain water, which may influence molecular dynamics of polysaccharide chains and be responsible for an appearance of the phase transition *T_w_* [77,85].

The theoretical curves, given in Figure 4, present the best fits to Equation (3) with respect to Equations (4) and (5) to the experimental data. The fitting parameters are collected in Table 3. The magnetization fraction corresponding to *T_1S_* relaxation times for all composites is relatively small and characterized by large scatter with numerous discontinuities, hence the analysis of molecular dynamics has been limited to *T_1L_*. The activation parameters, such as the pre-exponential factor *τ_0_*, the activation energy *E_a_*, and the distribution width of correlation times originating from molecular motions (hindered rotations of CH_3_ groups and local motions of polymer chain), were determined for pure PLLA, wood, and the PLLA/10 W/APE sample. For all PLLA/wood composites, a phase transition at about −82 °C was observed, the same as for the wood short component, therefore only the activation parameters for hindered rotation of CH_3_ groups were estimated. For the PLLA/20 W/APE and PLLA/30 W/APE composites, the minimum from CH_3_ groups was not observed. Therefore, wood used as a filler reduced the relaxation speed at low temperatures, hence slower relaxation occurred as confirmed by the estimated values of the *τ_0_* parameter. The *τ_0_* values, associated with motions of CH_3_ groups, ranged between 5.4 and 6.0 ps, while the E_a_ was between 10.7 and 11.1 kJ/mol. The obtained values indicated that the minimum corresponding to CH_3_ groups was shifted towards lower temperatures. The activation energies increased with increasing wood content, but remained lower when compared with neat PLLA. It implied an increase in the degree of crystallinity of PLLA/wood composites, which was also confirmed by WAXD analysis. The *β* parameter decreased for the composites when compared to neat PLLA for both the local polymer chain and CH_3_ groups’ movements. This indicated a greater distribution of activation parameters of the molecular movements. This outcome is fundamental for understanding the molecular level processes responsible for the industrially useful properties. For all composites, except for the PLLA/10 W/APE, a discontinuity in the course of relaxation times at a temperature of −82 °C (190 K) was observed and related to the phase transition observed in the course of the short relaxation time component of pure wood. Hence, the activation parameters could be obtained exclusively for the PLLA/10 W/APE sample. The activation energy *E_a_* of local motions of polymer chains in the system was equal to 58.2 kJ/mol, which is 1.5 times greater in comparison with pure PLLA [86,87]. This proves the restriction of molecular dynamics in the case of coated wood addition. At higher temperatures, the temperature dependency of relaxation times is similar for all tested samples. For samples with 20 and 30 wt %, the minimum at low temperatures was more flattened, and at high temperatures relaxation was slightly faster compared to pure PLLA. For the PLLA/wood/APE samples with 20 and 30 wt % of coupled wood, the low temperature minimum of CH_3_ groups was not obtained. It is supposed that reorientation of the CH_3_ groups was inhibited, hence the minimum was shifted towards lower temperatures, unattainable in the experiment.

The introduction of uncoated wood into PLLA caused changes in the dynamics of the investigated composites when compared to pure PLLA. In addition, the occurrence of three components of relaxation times for the PLLA/30 W sample proved poor compatibility between PLLA and the filler. The addition of non-modified wood accelerates crystallization of the PLLA chains when compared to neat PLLA as confirmed by longer relaxation times and lower activation energies [88]. The NMR results confirmed that using γ-aminopropyltriethoxysilane (APE) as a surface modifier improves interfacial adhesion between wood filler and PLLA. It is assumed that the reactive amino NH_2_ groups of APE interact strongly with PLLA chains, due to their capability of hydrogen bonding with PLLA. Modified wood through strong hydrogen bonds with PLLA chains influences molecular dynamics of PLLA chains. An increase in the activation energy E_a_, both for motions of CH_3_ groups and local polymer chains, can be explained by restricted molecular mobility in composites with the coupling agent [89,90,91].

**Figure 5:**
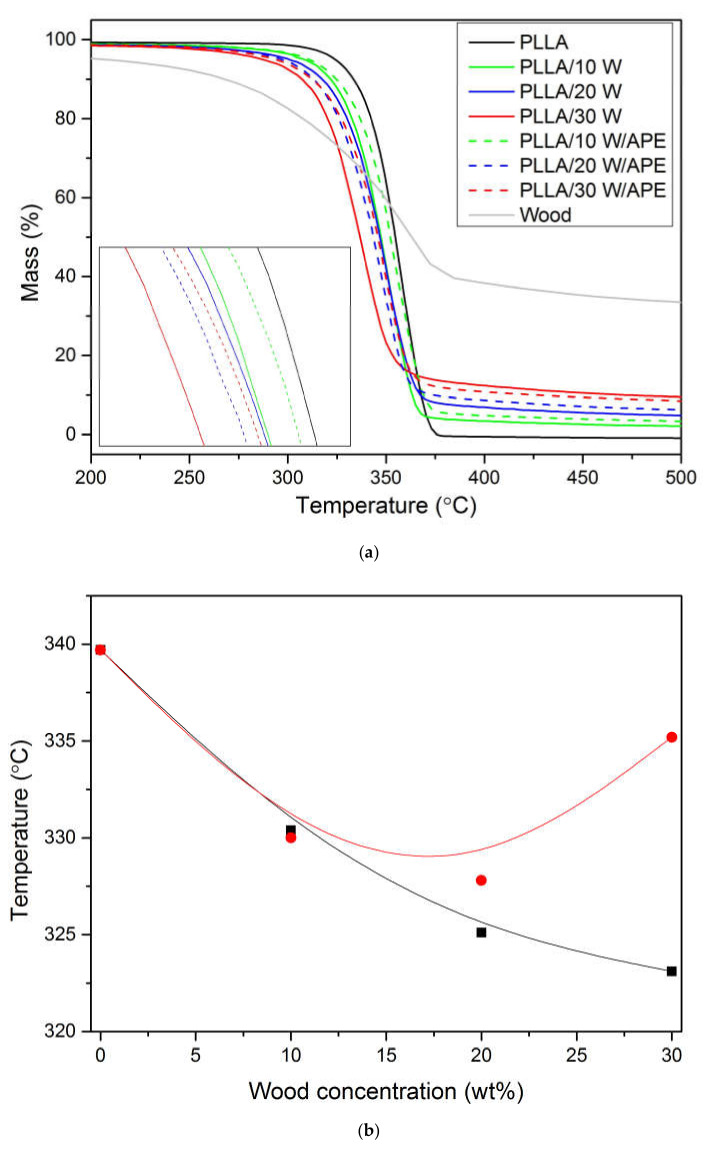
Raw TGA curves (**a**) and onset temperatures (*T_o_*) of PLA/wood composites in a function of wood concentration (**b**). Symbols: onset temperatures of PLA/wood (■) and PLA/wood/APE series (●). Lines were drawn to guide the eye.

Thermal stability of PLLA and wood-reinforced composites was determined using thermogravimetry analysis (TGA). Raw TGA curves were collected upon heating at 10 K min^−1^ and presented in Figure 5. All the tested samples revealed a single weight loss step. Pure PLLA thermogram exhibited no significant weight loss until approximately 340 °C. The major degradation step was finalized at 374 °C, where no residue remained. The mechanism of PLLA thermal degradation implies scission of two linkages: carbonyl carbon-oxygen and the carbonyl carbon–carbon linkage [92]. Decomposition of wood, instead, starts with the loss of hemicellulose and cellulose, followed by lignin [93]. All these processes overlap in the analyzed composites, resulting in a single step degradation shifted towards lower temperatures in comparison with neat PLLA. In the case of PLLA/wood composites, a gradual decrease of the onset temperature (*T_o_*) as a function of wood content can be noticed. The lower thermal stability of the composites was expected, taking into account that wood has a lower thermal stability in comparison to PLLA, as also seen in Figure 5 [94]. Degradation of the APE-compatibilized composites starts at higher temperatures compared to non-compatibilized counterparts, probing their higher thermal stability (Figure 5b). Compatibilization causes an increase in the dissociation energy needed for scission of newly created chemical bonds, which results in enhanced thermal stability of the APE-based formulations. In addition, it favors dispersion of the filler, which enhances thermal stability of the entire system [95].

For the wood-reinforced composites, an ash line proportional to filler content, not found in the pure PLLA curve, was identified (Figure 5). The actual residual mass values (*m_R_*) are summarized in Table 2. Taking into account large filler content and the fact that approximately 30 wt % of the initial filler mass remained after its decomposition, it was expected that the addition of the wood filler into PLLA would result in an increase in residual mass value. This proportional increase is independent of the compatibilization process proposed in the study. The formation of residual mass is a result of carbide formation during filler decomposition [95].

**Figure 6:**
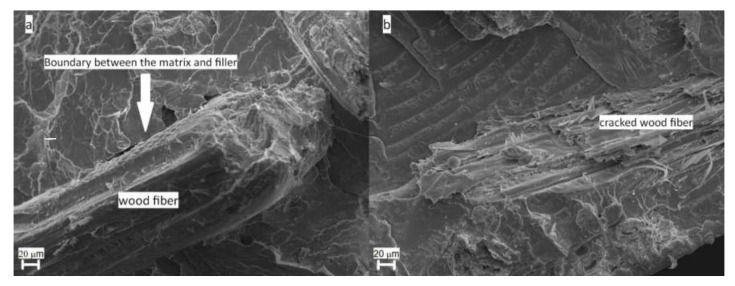
SEM micrographs of PLLA/10 W (**a**) and PLLA/10 W/APE (**b**) (800×).

To gain information about the phase structure and morphology of the PLLA composites containing wood and compatibilizer, SEM analyses were performed. The selected results are presented in Figure 6. The surface of plain PLLA, not presented herein, appears quite smooth as expected. Wood particles, as well reported in the literature [12,16,96], reveal quite a smooth surface with clearly visible fiber-like structure consisting of different strands’ bundles. Morphology of the PLLA composite containing 10 wt % of wood is presented in Figure 6a. The micrograph presents a filler particle pulled out and separated from the PLLA matrix, with no polymer traces on the wood surface. Wood particles reveal quite a smooth surface, thus it is difficult to achieve sufficient interfacial adhesion via mechanical interlocking [96] or chemical bonding due to different polarities of components. Poor adhesion in turn results in the creation of voids on the filler–matrix borders, clearly seen in Figure 6a, which result in premature failure of the entire system as proven in [25,26]. The fractured surface morphology changes drastically upon the addition of the APE compatibilizer, as depicted in Figure 6b. Coated wooden particles well adhere to the PLLA matrix due to enhanced connection between the matrix and filler. Long APE alkyl chains are covalently and hydrogen bonded to the wood surface via silane, which enhances filler hydrophobicity and therefore adhesion between the filler and matrix [11,97,98]. Moreover, it is evident that material failure proceeds throughout the wooden particle while connection between the filler and matrix is unaffected by the external load applied during the breakthrough. These significant changes in morphology are expected to reflect in mechanical properties of the blends.

**Figure 7:**
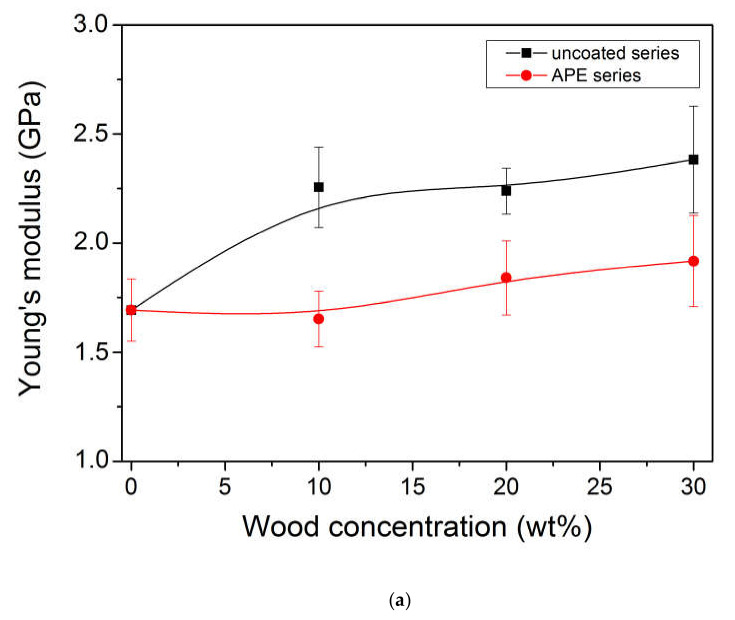

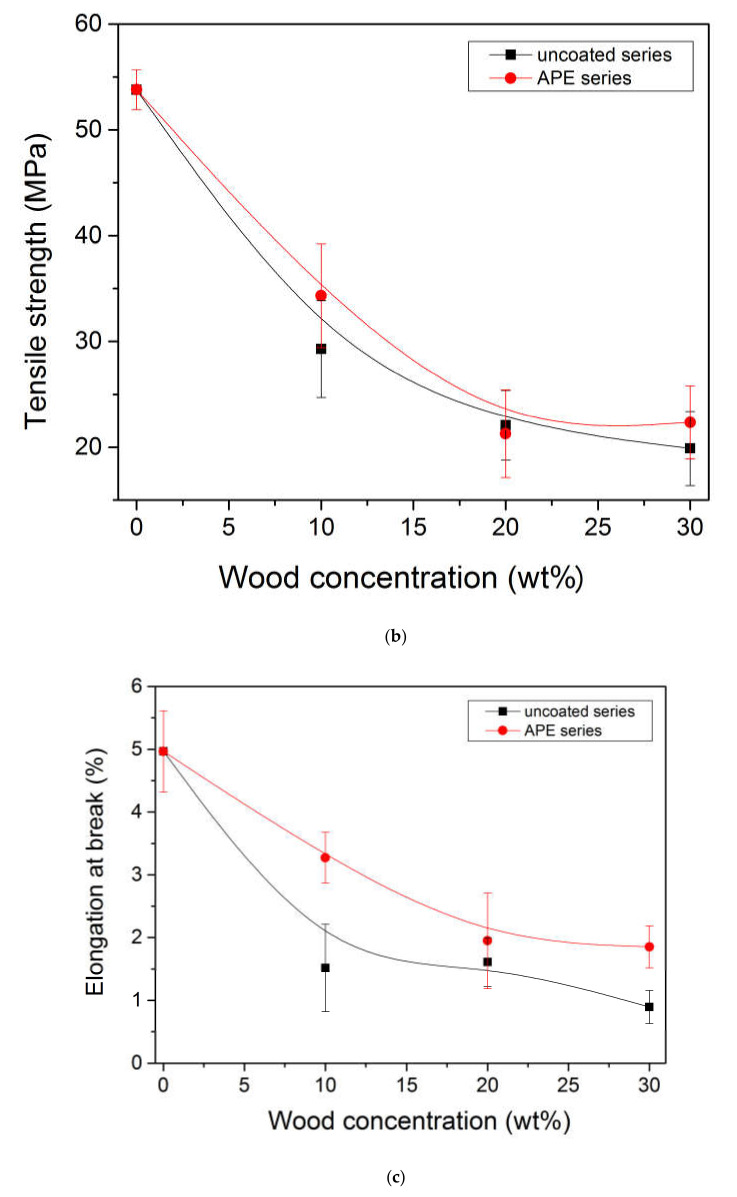

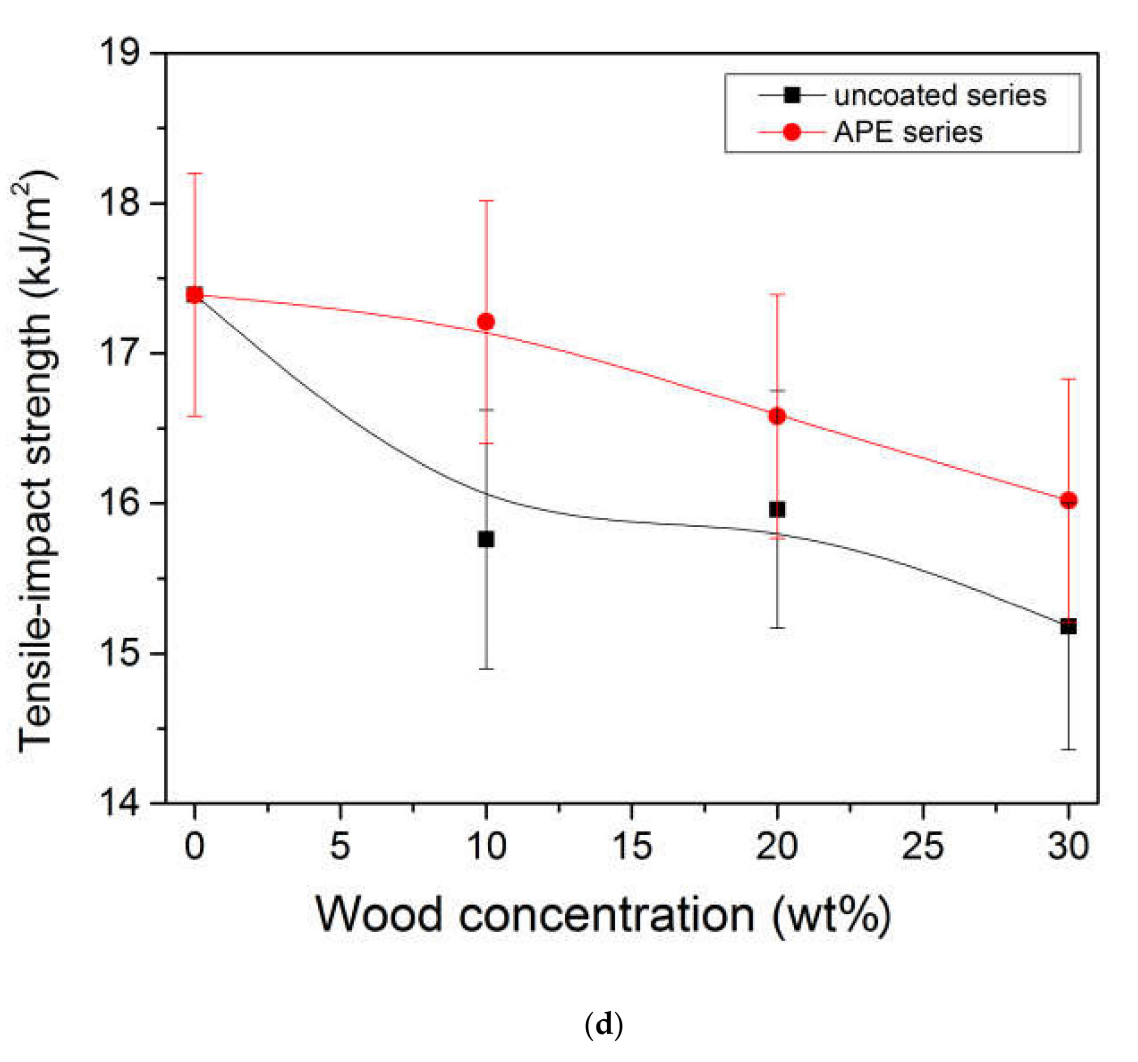
Effect of wood concentration and filler treatment on stiffness (**a**), tensile strength (**b**), elongation at break (**c**), and tensile impact strength (**d**) of PLLA/wood composites. Symbols: PLLA/wood (■) and PLLA/wood/APE (●).

Mechanical properties of PLLA/wood and PLLA/wood/APE composites are presented in Figure 7a–d. Figure 7a–c represents tensile properties of PLLA formulations. All PLLA-based composites exhibited brittle fracture, in agreement with literature data [3,4]. Pure PLLA revealed an elongation at break of 5% and Young’s modulus of 1.7 GPa. Upon addition of uncoated wood, stiffening of the system was evidenced by increased Young’s modulus and decreased elongation at break values. In the case of the PLLA/wood/APE samples, the elastic modulus was lower comparing with the PLLA/wood composite, indicating somehow a lubrication effect of the APE layers, created on the surface between wood particles and the polymeric matrix. The tensile modulus, which is measured at very low elongations, utilizes a lower physical interaction appearing between the polymeric matrix and wood filler due to the presence of the APE layer. This phenomenon may lead to easier displacement of these two phases in the initial phase of elongation and should be taken into consideration. The varied trend of modulus with composition may also confirm an improved dispersion of the filler, promoted by APE coating of pine wood particles.

The filler addition into PLLA-based formulations caused a decrease in tensile strength (Figure 7b), in a function of filler content, with slightly higher values, although close to experimental error, noted for the compatibilized series. One of the key factors in terms of strengthening fiber-filled composites is the aspect ratio of the applied filler, which determines further mechanical properties, especially tensile strength. In the case of the PLLA/wood composites, a very complex mixture of rather short fibers was used. In such a case, fibers are pulled out from the matrix upon deformation, as also evidenced by the SEM technique, resulting in poor tensile properties of the composite [99]. Effective compatibilization is supposed to reflect in enhanced tensile strength of the obtained composites as large deformations affect mobility of the interphase. This is not the case in PLLA/wood/APE composites, as the observed changes were rather close to experimental error. Thus, it was concluded that although a chemical interaction between the components appeared, as reported above, it was not sufficient to significantly affect the behavior of the composites in extent that could be significant from the application point of view.

The tensile impact strength results, presented in Figure 7d, revealed a decreasing tendency, from 17.5 down to 15 kJ/m^2^ for all the filled composites together with wood content, as frequently observed for the composites of thermoplastic matrix and particles with dimensions comparable as in our case. Bonding in such systems is limited, hence the addition of uncoated wood particles induces the formation of voids at the wood/polymer boundary, which in turn causes the composite to crack easier under external stresses [1,16]. Worth noting is the fact that PLLA/wood/APE composites revealed somehow higher impact strength in comparison with non-modified series, hence the system is more resistant to dynamic external loads.

Surface treatment of the filler, such as APE coating used in the study, is widely exploited to influence interactions in highly filled polymers [100]. The main aim of the process is to reduce high surface energy of the filler, which in turn leads to decreased aggregation, improved homogeneity, ease of processing, and better surface quality. Lowering of the surface tension of the filler also decreases the matrix/filler interaction and results in decreased yield stress and strength. However, in the case of aminofunctional silanes, which exhibit intense reactivity, this effect is compensated by the assumed strong covalent bonding created between the coupling agent and the polymer matrix [100]. This interaction, confirmed in the study by the SEM and NMR results presented above, is expected to reflect in better deformability of the PLLA/wood/APE composite, provided that silanization is refined further [33,101].

## 4. Conclusions

Composites prepared with PLLA and pine wood, both bio-based, have been produced by twin-screw extrusion in different compositions, from 10 to 30 wt % of filler content, and characterized for their morphology, physicochemical and mechanical properties. Impregnation of pine wood with γ-aminopropyltriethoxysilane can improve adhesion between the phases, with positive effects on material properties. The improved morphology was proven by SEM. Analysis of relaxation times by NMR indicated higher compatibility between the filler and matrix in case of surface-modified formulations. Molecular dynamics analysis proves restriction in mobility of polymer chains and CH_3_ side groups in case of APE-modified samples. In the non-compatibilized composites, the activation energies of CH_3_ groups are lower. Thermogravimetric analysis revealed that wood addition causes a decrease in thermal stability of the material, which occurred to a lower extent in the compatibilized formulations. Studies on mechanical properties indicated that the uncoated filler addition results in higher stiffness of the composites, wherein wood compatibilization leads to an enhanced connection between the matrix and filler and results in greater deformability of the entire system. Worth noting is the fact that although enhanced connection between the PLLA/wood/APE components is evident, the silanization process still needs to be improved in order to obtain satisfactory mechanical behavior that can be tolerated by practice.

## Figures and Tables

**Table 1:** Symbols and mass concentrations of samples.

Designation	Mass Concentration (wt %)		
	PLLA	Wood	Compatibilized Wood
PLLA	100	0	0
PLLA/10 W	90	10	0
PLLA/20 W	80	20	0
PLLA/30 W	70	30	0
PLLA/10 W/APE	90	0	10
PLLA/20 W/APE	80	0	20
PLLA/30 W/APE	70	0	30

**Table 2:** Cold crystallization (*T_cc_*), melting (*T_m_*) and crystallization (*T_c_*) temperatures, onset of glass transition (*T_g_*), melting enthalpy (*ΔH_m_*), crystallinity degree (*X_c_*), and residual mass (*m_R_*).

Sample	*T_g_* (°C)	*T_cc_* (°C)	*T_m_* (°C)	ΔH_m_ (J/g)	*T_c_* (°C)	*X_c_* (%)	*m_R_* (%)
PLLA	67	98	177	55	102	5	0
PLLA/10 W	67	93	178	65	110	7	2
PLLA/20 W	66	90	174	62	111	11	4
PLLA/30 W	65	89	175	60	111	12	8
PLLA/10 W/APE	67	94	177	56	107	7	3
PLLA/20 W/APE	66	90	175	57	111	14	5
PLLA/30 W/APE	66	89	175	59	112	14	7

**Table 3:** Motional parameters obtained for PLLA formulations from fitting the theoretical relaxation curves to experimental data. The values of uncertainty of the estimated parameters were lower than 10%. T_1L_ is the long and T_1S_ is short relaxation time, *τ_0_* is the pre-exponential factor, *E_a_* is the activation energy of molecular motions, and *β* is the distribution width of correlation times.

		Hindered Rotation of Methyl Groups CH_3_ Around Their Axes Symmetries			Local Motions of Polymer Chains		
Sample	*T_1L_/T_1S_*	τ_0_ (s)	*E_a_* (kJ/mol)	*β*	*τ_0_* (s)	*E_a_* (kJ/mol)	*β*
PLLA	T_1L_	5.3 × 10^−13^	12.5	0.4	2.1 × 10^−16^	37.3	0.2
PLLA	T_1S_	4.8 × 10^−12^	13.9	0.4	4.9 × 10^−16^	33.3	0.2
PLLA/10 W	T_1L_	5.4 × 10^−12^	10.7	0.2	**N/A**		
PLLA/20 W	T_1L_	5.9 × 10^−12^	10.0	0.2	**N/A**		
PLLA/30 W	T_1L_	6.0 × 10^−12^	11.1	0.2	**N/A**		
PLLA/10 W/APE	T_1L_	8.4 × 10^−13^	14.1	0.2	3.5 × 10^−16^	58.2	0.1
PLLA/20 W/APE	T_1L_	**N/A**			**N/A**		
PLLA/30 W/APE	T_1L_	**N/A**			**N/A**		
Wood *	T_1L_	8.2 × 10^−11^	6.7	0.3	2.2 × 10^−12^	22.4	0.6

* In the case of wood the first set of parameters is associated with segmental motions of polysacharide chains and the second with local chains’ motion.
